# Sickness absence among employees of healthcare organizations in the public sector in Mongolia: A cross‐sectional study

**DOI:** 10.1002/1348-9585.12390

**Published:** 2023-02-28

**Authors:** Erdenetuya Sukhee, Tugsdelger Sovd, Ulzii‐Orshikh Khaltar, Nobuyuki Hamajima, Enkhbold Sereenen, Narantuya Davaakhuu, Eiko Yamamoto

**Affiliations:** ^1^ Department of Healthcare Administration Nagoya University Graduate School of Medicine Nagoya Japan; ^2^ Department of Public Administration and Management Ministry of Health Ulaanbaatar Mongolia; ^3^ Department of Monitoring and Evaluation and Internal Audit Ministry of Health Ulaanbaatar Mongolia; ^4^ The Digital Development and Marketing Department Mongolian National University of Medical Sciences Ulaanbaatar Mongolia; ^5^ Medicine and Medical Devices Regulatory Agency Ulaanbaatar Mongolia

**Keywords:** absenteeism cost, healthcare workers, Mongolia, sickness absence

## Abstract

**Objectives:**

This study aimed to understand the rate of sickness absence (SA) among employees of public healthcare organizations in Mongolia, to identify factors associated with long‐term SA, and to estimate costs due to SA.

**Methods:**

This cross‐sectional study included employees of public healthcare organizations who had certified SA from 2016 to 2018. Sociodemographic and occupational characteristics of absentees and the data on absences were collected. A logistic regression analysis was performed to identify factors associated with long‐term SA (≥15 days) among employees who had SA. Absence parameters and the average costs due to SA were calculated and the total cost due to SA at all public healthcare organizations was estimated.

**Results:**

From 2016 to 2018, there were 13 653 absentees and 21 043 SA, and the absence rate was 0.9%. The average absence length per absence and absentee were 9.63 days and 14.85 days, respectively. Factors associated with long‐term SA were age ≥40 years, 10–19 years in employment, working at the second and tertiary levels, and night shift. The average cost per absentee was 295.5 USD, and the estimated total cost for all health organizations was 1 796 993 USD per year.

**Conclusions:**

The absence rate was 0.9% and older age, longer work experience, higher organizational level, and night shift were associated with taking a long‐term SA. To reduce the costs of absenteeism and promote the health of employees in healthcare organizations, policymakers should review the policies related to SA and develop national guidelines on SA for employers, healthcare managers, and employees.

## 1 INTRODUCTION

Sickness absence (SA) is one of the main concerns related to human resource management in all countries. Health facilities are a high‐risk environment for healthcare workers (HCWs) to have infections and other diseases because of tough working conditions.^1^, ^2^ Previous studies reported that 41.4%–95% of HCWs had worked while they had symptoms of an acute respiratory illness or influenza‐like illness.^3^, ^4^, ^5^ At healthcare facilities, not only disease progression of sick HCWs but also risk of disease transmission from sick HCWs to patients should be prevented. Disease transmission to other HCWs and absences of HCWs due to severe illness negatively affect work efficiency and increase the economic burden of HCWs and healthcare facilities. A high rate of absence of workers reduces the productivity of workers and organizations, as well as increases costs due to SA. Moreover, a previous study suggested that workers who had an SA longer than 15 days per year had a 3.7–4.7 times higher risk of mortality due to all causes, including cancers, cardiovascular diseases, alcohol‐related diseases, and suicides.^6^ Therefore, it is important to understand the characteristics of HCWs who take long‐term SA to reduce hazards at work and to improve workplace conditions.

Most studies on SA have been conducted in developed countries and there is less evidence on SA in developing countries, where a shortage of HCWs is one of the major problems for providing health service to the population in developing countries.^7^, ^8^ Mongolia is a lower middle‐income country located in East Asia with a population of 3.41 million. There has been only one study on sickness absenteeism in Mongolia, which included 1330 employees in private companies.^9^ The study focused on absences due to illness of the employees or their families related to air pollution in winter and factors associated with wintertime absences were females, having a child, and more years in employment. It was also suggested that the total cost due to wintertime absences might be fairly large. However, to the best of our knowledge, there have been no studies on SA among HCWs in Mongolia. Mongolia faces problems with human resources in its health sector, such as a shortage of nurses and maldistribution of HCWs between urban and rural areas as well as between primary and referral levels of health facilities.^10^, ^11^, ^12^ It is suggested that a shortage of HCWs increases the workload, stress at workplaces, fatigue, and illness among HCWs.^13^, ^14^ Frequent SA leads to understaffing and the training of replacement personnel is often costly and time‐consuming. Therefore, this study aimed to identify the frequency of SA and factors associated with long‐term SA among HCWs who had SA in the public sector in Mongolia and to estimate costs and productivity loss due to SA.

## 2 METHODS

### 2.1 Study design

In 2019, the Mongolian Ministry of Health (MoH) sent a questionnaire to all 434 healthcare organizations in the public sector and all 219 family health centers (FHCs) to collect the data of all SA of HCWs from 2016 to 2018, which were based on the sick leave certificates issued by doctors. Absences due to maternity leave, diagnoses related to conditions originating in the perinatal period (ICD10 code‐16), and diagnoses related to congenital anomalies, deformities, and chromosomal abnormalities (ICD10 code‐17) were excluded. There were 434 healthcare organizations in the public sector that were registered to the MoH: ten hospitals and six centers at the national level, five regional diagnostic and treatment centers, 16 provincial hospitals, two general hospitals and eight health centers of the district level, 292 health centers and 45 hospitals of the subprovince and village level, 22 health department offices (Ulaanbaatar City and 21 provinces), and 28 other healthcare organizations (three maternity hospitals, one emergency center, one narcology center, one stomatology center, one hospital for poor people, 14 zoonosis centers, and seven traditional medical centers). FHCs, which are privately managed, were included in the survey because the property and funding are from the state budget, and FHCs provide primary healthcare by contracting with local governments. In this study, HCWs were defined as workers who worked for healthcare organizations, including not only medical professionals but also administrative and nonmedical support staff. A total of 282 facilities, including 193 healthcare organizations in the public sector and 89 FHCs answered the questionnaire and the response rate was 43.2%.

### 2.2 Medical certificate of sick leave

The procedure of issuing a medical certificate for sick leave and compensation was approved by the Minister of Labor and Social Welfare. The process is regulated mainly by the law on pensions and benefits from the social insurance fund. According to related regulations, a medical certificate for sick leave shall be issued based on the medical record and reviewed by a deputy director who is in charge of medical care or commission of medical quality control. Certificates that are submitted to social insurance organizations are checked by an inspector of social insurance. According to the law, compensation for sick leave is 50%, 55%, and 75% of the wage for workers whose years in employment are less than 5 years, 5–14 years, and 15 years or longer, respectively. The duration of sick leave is decided by a doctor who issues a certificate but not regulated by the length of employment. The minimum length of sick leave is 1 day. The employer (healthcare organization) pays compensation for the first 5 days of sick leave and the rest is paid by the social insurance fund. The template of a medical certificate for sick leave includes name, personal ID, age, gender, home address, workplace, position, diagnosis by ICD‐10, the first and last dates of the sick leave, and the amount of compensation by the employers and by the social insurance fund. Absence days of the sick leave were calculated as the difference between the first and last dates of the sick leave.

### 2.3 Absence parameters

The number of employees at healthcare organizations was taken from the database of the Center for Health Development and the Ulaanbaatar City Health Department. The total number of HCWs at all healthcare organizations and the 282 organizations that responded was 28 976 and 21 285 in 2016, 29 572 and 22 115 in 2017, and 29 903 and 22 828 in 2018, respectively.

The absence rate (AR) was calculated as follows^15^, ^16^:

$$\frac{\text{The total number of absence days}\times 5/7}{\text{The total available workdays of}\;\mathrm{all}\;\text{employees}\;\mathrm{at}\;\text{responded organizations}}\times 100.$$

Absence days were calendar days and converted into lost workdays by dividing by seven and multiplying five because available workdays did not include weekends (Appendix). The absence frequency rate (FR) refers to the average number of absences per employee as a percentage. FR was calculated by dividing the total number of absences by the total number of employees at responded organizations and multiplying by 100.^16^ Descriptive analysis was performed to calculate the average and 95% confidence interval (CI) of absence length, absence length per absentee, absence length per employee, and frequency of absences per absentee.

### 2.4 Data of sickness absence

The data of SA from 2016 to 2018 were collected using a questionnaire that included such information as absence information (diagnosis by ICD‐10 code, duration, the first and the last dates, and the amount of compensation), sociodemographic information of absentees (gender, age, marital status, number of children, and housing type), and work‐related information of absentees (healthcare organization, name of department, position, shift work, and employment years). The data of 21 133 sick leave certificates of 13 715 absentees were collected, but 90 certificates were excluded because of maternity leave (*n* = 5), diagnosis related to conditions originating in the perinatal period (ICD10 code‐16, *n* = 34), and diagnosis related to congenital anomalies, deformities, chromosomal abnormalities (ICD10 code‐17, *n* = 51). Finally, 21 043 sick leaves of 13 653 absentees were included in this study. According to the last dates of SA, SA in each year was decided.

### 2.5 Variables of absentees and absences

Marital status was categorized into single/divorced or married. Housing types were categorized into three groups (own house/apartment room, rental room/dormitory, or Mongolian traditional ger). For calculation of the average salary of each absentee, occupations were divided into five groups based on the kind of job: (1) medical doctor and dentist, (2) nurse, midwife, and feldsher (medical assistant), (3) other medical and public health professional, (4) administrative and nonmedical support staff, and (5) director and manager. In descriptive and logistic regression analyses, three groups of occupations were used (nurse/midwife/feldsher, other medical and public health professional, or nonmedical worker/director/manager). The levels of healthcare organizations were categorized into four categories: primary (FHCs, health centers, and hospitals at the sub‐provincial and village level), secondary (hospitals and health centers, maternity hospitals, emergency, narcology, zoonosis, and traditional medical centers in the district and provincial level, regional diagnostic and treatment centers), tertiary (national hospitals and specialized centers), or health department (provincial health departments, the Ulaanbaatar City Health Department, and the Center for Health Development). The group of health departments included organizations that have an administrative function but do not provide medical services. A repeat of SA refers to taking two SA or more in a year. Areas of healthcare organizations were categorized into urban or rural according to the location.

### 2.6 Statistical analysis

In this study, short‐term and long‐term SA were defined as the length of absence ≤14 days and ≥15 days in a year, respectively, based on the average absence length per absentee (14.8 days).^17^, ^18^ A logistic regression analysis was used to identify factors associated with long‐term SA among HCWs who had SA. Statistical Package for Social Sciences version 26 (IBM SPSS Inc., New York, USA) was used for data analysis.

### 2.7 Estimation of costs due to absenteeism.

Data on the salary of employees at the 282 healthcare organizations from 2016 to 2018 were used to estimate the costs due to SA, such as the total lost salary, the total cost of loss of productivity, and the total cost due to SA. The data were extracted from the Financial Department of the MoH, including employee's name, position, years in employment, job level, salary grade, amount of base salary, all remuneration, and total salary. A bottom‐up costing approach was used for the calculation of costs due to SA.^19^, ^20^ This method is based on estimating the lowest possible level of work costs and then the unit costs are aggregated to estimate the total costs.

First, the average revenue per employee in each year (the total revenue of an organization/the total number of employees of the organization) and the average revenue per employee per day (the average revenue per employee per year/the number of available workdays per year) were estimated using the data of 13 653 absentees. Next, the average salary of each absentee per month was calculated according to the five occupational groups (total salary of employees in the group of the healthcare organization/total number of employees in the group of the healthcare organization). The average salary of absentees per day was calculated by dividing the average salary of absentees per month by the available workdays per month. Subsequently, lost productivity (average revenue per day × AR of absentee) and lost salary (average salary per day × AR of absentee ‐ average compensation) of each absentee were calculated. Average compensation was used because the average salary of the five occupational groups was used to calculate the lost salary. The average compensation of an absentee was calculated by multiplying the lost salary by the weighted average with percentage (62.65%). The weighted average was calculated as the sum of weights who received 50%, 55%, and 75% compensation. Finally, the total cost (lost salary + lost productivity + total compensation) per absentee was calculated, and the total cost was estimated by summing the total cost of all absentees from 2016 to 2018.

## 3 RESULTS

From 2016 to 2018, there were 13 653 absentees and 21 043 SA with a total of 202 716 absence days (Table 1). The number of absentees accounted for 19.7%–21.5% of the total number of employees. The AR was 0.9% (range, 0.8–0.9), and the absence FR was 31.8% (range, 29.7%–33.3%) during the 3 years. The length of absence ranged from 1 to 90 days, and the average length of absence was 9.63 days (95% CI 9.55–9.71). The absence length per absentee ranged from 1 to 226 days and the average was 14.85 days (95% CI 14.60–15.10). The average absence length per employee was 3.06 days (95% CI 3.05–3.07), which means that each employee lost 3.06 workdays in a year. The number of absences per absentee ranged from 1 to 13, and the average frequency was 1.54 (95% CI 1.52–1.56). There were no differences in each absence parameter in the 3 years.

**TABLE 1:** Parameters of sickness absences at 282 healthcare organizations from 2016 to 2018.

Variables	2016	2017	2018	Total
Total number of employees	21 285	22 115	22 828	66 228
Available workdays (days)	247	249	252	748
Total available workdays of all employees (days)	5 257 395	5 506 635	5 752 656	16 516 686
Number of absentees (%)	4195 (19.7%)	4745 (21.5%)	4713 (20.6%)	13 653 (20.6%)
Number of absences	6320	7356	7367	21 043
Total absence days	61 050	70 402	71 264	202 716
Absence rate (%)	0.8	0.9	0.9	0.9
Absence frequency rate (%)	29.7	33.3	32.3	31.8
Average absence length (days) [95% CI]	9.66 [9.50–9.82]	9.57 [9.44–9.71]	9.67 [9.54–9.82]	9.63 [9.55–9.71]
Average absence length per absentee (days) [95% CI]	14.55 [14.13–15.03]	14.84 [14.39–15.28]	15.12 [14.65–15.59]	14.85 [14.60–15.10]
Average absence length per employee (days) [95% CI]	2.87 [2.85–2.89]	3.18 [3.16–3.21]	3.12 [3.10–3.14]	3.06 [3.05–3.07]
Average frequency of absences per absentee [95% CI]	1.51 [1.48–1.54]	1.55 [1.52–1.58]	1.56 [1.53–1.59]	1.54 [1.52–1.56]

Abbreviation: CI, confidence interval.

To understand the characteristics of the sick leaves of HCWs, the data of 21 043 sick leaves of 13 653 absentees were analyzed. Of the total 21 043 absences, the most common diagnosis was diseases of the genitourinary system (*n* = 3436, 16.3%), followed by diseases of the digestive system (*n* = 2421, 11.5%), diseases of the circulatory system (*n* = 2381, 11.3%), injury, poisoning and certain other consequences of external causes (*n* = 2283, 10.8%), and diseases of the nervous system (*n* = 1991, 9.5%) (Table 2). When the average length of absence was compared among the top 10 diagnoses, the absence length due to neoplasm was the longest (14.05 days) followed by injury, poisoning, and certain other consequences of an external cause (13.55 days), and certain infectious and parasitic diseases (12.89 days).

**TABLE 2:** The absence frequency and the average length of absence according to top 10 ICD‐10 diagnostic categories from 2016 to 2018 (*n* = 21 043).

ICD‐10 diagnostic category	Absence *n* (%)	Average absence length (days) [95% CI]
Diseases of the genitourinary system	3436 (16.3%)	8.71 [8.58–8.86]
Diseases of the digestive system	2421 (11.5%)	8.53 [8.34–8.73]
Diseases of the circulatory system	2381 (11.3%)	9.07 [8.88–9.28]
Injury, poisoning and certain other consequences of external causes	2283 (10.8%)	13.55 [13.19–13.93]
Diseases of the nervous system	1991 (9.5%)	8.97 [8.77–9.17]
Diseases of the respiratory system	1726 (8.2%)	7.58 [7.39–7.78]
Diseases of the musculoskeletal system and connective tissue	1232 (5.9%)	9.31 [9.04–9.59]
Neoplasms	937 (4.5%)	14.05 [13.53–14.56]
Certain infectious and parasitic disease	720 (3.4%)	12.89 [12.21–13.70]
Diseases of the skin and subcutaneous tissue	609 (2.9%)	8.72 [8.38–9.09]

Abbreviations: CI, confidence interval; ICD‐10, International Classification of Diseases 10th Revision.

Next, the socidemographic and occupational characteristics of 13 653 absentees were analyzed (Table 3). Most absentees were females (89.6%), 39 years old or younger (45.8%), married (82.7%), having children (91.6%), and living in their own house or apartment (63.5%). In terms of occupational factors, most absentees were nurses/midwives/feldshers (40.2%), worked for the secondary level of healthcare organizations (52.6%), had 9 years or less in employment (42.8%), and had night shift (52.3%). Of all absentees, 4453 absentees (32.6%) repeated SA within a year. The characteristics of absentees in each year were almost the same.

**TABLE 3:** Sociodemographic and occupational characteristics of absentees (*n* = 13 653).

Variables	2016 (*n* = 4195)	(2017 *n* = 4745)	2018 (*n* = 4713)	Total (*n* = 13 653)
	*n* (%)	*n* (%)	*n* (%)	*n* (%)
*Gender*				
Male	453 (10.8)	488 (10.3)	475 (10.1)	1416 (10.4)
Female	3742 (89.2)	4257 (89.7)	4238 (89.9)	12 237 (89.6)
*Age group (years old)*				
≤39	1929 (46.0)	2118 (44.6)	2211 (46.9)	6258 (45.8)
40–49	1401 (33.4)	1545 (32.6)	1436 (30.5)	4382 (32.1)
50≤	865 (20.6)	1082 (22.8)	1066 (22.6)	3013 (22.1)
*Marital status*				
Single/divorced	777 (18.5)	782 (16.5)	804 (17.1)	2363 (17.3)
Married	3418 (81.5)	3963 (83.5)	3909 (82.9)	11 290 (82.7)
*Number of children*				
0	304 (7.2)	403 (8.5)	444 (9.4)	1151 (8.4)
1–2	2708 (64.6)	2968 (62.2)	2993 (63.5)	8669 (63.5)
3≤	1183 (28.2)	1374 (29.0)	1276 (27.1)	3833 (28.1)
*Housing type*				
Own house/apartment	2688 (64.1)	3038 (64.0)	2948 (62.6)	8674 (63.5)
Rental/dormitory	234 (5.6)	251 (5.3)	247 (5.2)	732 (5.4)
Traditional ger	1273 (30.3)	1456 (30.7)	1518 (32.2)	4247 (31.1)
*Occupation*				
Nurse/midwife/feldsher	1690 (40.3)	1887 (39.8)	1910 (40.5)	5487 (40.2)
Other medical	1275 (30.4)	1463 (30.8)	1431 (30.4)	4169 (30.5)
Non‐medical/director/ manager	1230 (29.3)	1395 (29.4)	1372 (29.1)	3997 (29.3)
*Level of health organization*				
Primary	564 (13.4)	660 (13.9)	646 (13.7)	1870 (13.7)
Secondary	2179 (52.0)	2466 (52.0)	2540 (53.9)	7185 (52.6)
Tertiary	1363 (32.5)	1525 (32.1)	1435 (30.4)	4323 (31.7)
Health department	89 (2.1)	94 (2.0)	92 (2.0)	275 (2.0)
*Years in employment (years)*				
≤9	1757 (41.9)	1979 (41.7)	2107(44.7)	5843 (42.8)
10–19	1028 (24.5)	1155 (24.3)	1168 (24.8)	3351 (24.5)
20≤	1410 (33.6)	1611 (34.0)	1438 (30.5)	4459 (32.7)
*Night shift*				
No	1964 (46.8)	2316 (48.8)	2237 (47.5)	6517 (47.7)
Yes	2231 (53.2)	2429 (51.2)	2476 (52.5)	7136 (52.3)
*Repeat of sickness absence*				
No	2883 (68.7)	3214 (67.7)	3103 (65.8)	9200 (67.4)
Yes	1312 (31.3)	1531 (32.3)	1610 (34.2)	4453 (32.6)

To identify the factors associated with having a long‐term absence among HCWs who had SA, all 13 653 absentees were divided into two groups based on the average length of absence. There were 9844 absentees (72.1%) who had 14 absence days or less in a year (the short‐term group) and 3809 absentees (27.9%) who had 15 absence days or more in a year (the long‐term group). In binary logistic regression analysis, absentees who were 40–49 years old (odds ratio (OR) = 1.25, 95% CI 1.15–1.36) and 50 years old or older (OR = 1.37, 95% CI 1.25–1.51) than 39 years old or younger, worked for the secondary level (OR = 1.17, 95% CI 1.04–1.31) and the tertiary levels (OR = 1.34, 95% CI 1.19–1.52) than the primary level, worked for 10–19 years (OR = 1.24, 95% CI 1.12–1.36) and for 20 years or longer (OR = 1.33, 95% CI 1.22–1.45) than for less than 10 years, and had night shift work (OR = 1.14, 95% CI 1.06–1.23) had significantly more long‐term SA (Table 4). Compared with nurses, midwives, or feldshers, the other medical professionals had significantly less long‐term SA (OR = 0.88, 95% CI 0.80–0.97). In multivariate analysis, including all sociodemographic and work‐related variables, the age groups of 40–49 years old (OR = 1.17, 95% CI 1.04–1.31) and 50 years old or older (OR = 1.30, 95% CI 1.12–1.49), working for the secondary level (OR = 1.15, 95% CI 1.02–1.30) and the tertiary level (OR = 1.31, 95% CI 1.15–1.49) of healthcare organizations, 10–19 years in employment (OR = 1.14, 95% CI 1.02–1.27) and night shift (OR = 1.13, 95% CI 1.04–1.23) were significantly associated with having long‐term SA.

**TABLE 4:** Factors associated with having long‐term sickness absences (*n* = 13 653).

Variables	Short‐term	Long‐term	OR (95% CI)	AOR^a^ (95% CI)
	*n* (%)	*n* (%)		
*Gender*				
Male	1029 (72.7)	387 (27.3)	1 (Reference)	1 (Reference)
Female	8815 (72.0)	3422 (28.0)	1.03 (0.91–1.17)	1.02 (0.89–1.15)
*Age group (years old)*				
≤39	4690 (74.9)	1568 (25.1)	1 (Reference)	1 (Reference)
40–49	3090 (70.5)	1292 (29.5)	1.25 (1.15–1.36)***	1.17 (1.04–1.31)*
50≤	2064 (68.5)	949 (31.5)	1.37 (1.25–1.51)***	1.30 (1.12–1.49)***
*Marital status*				
Single/divorced	1713 (72.5)	650 (27.5)	1 (Reference)	1 (Reference)
Married	8131 (72.0)	3159 (28.0)	1.02 (0.93–1.13)	1.02 (0.91–1.13)
*Number of children*				
0	857 (74.5)	294 (25.5)	1 (Reference)	1 (Reference)
1–2	6231 (71.9)	2438 (28.1)	1.14 (0.99–1.31)	1.00 (0.85–1.16)
3≤	2756 (71.9)	1077 (28.1)	1.14 (0.98–1.32)	0.93 (0.78–1.10)
*Housing type*				
Own house/apartment	6233 (71.9)	2441 (28.1)	1 (Reference)	1 (Reference)
Rental/dormitory	546 (74.6)	186 (25.4)	0.87 (0.73–1.03)	0.91 (0.77–1.09)
Traditional ger	3065 (72.2)	1182 (27.8)	0.98 (0.91–1.07)	0.99 (0.91–1.08)
*Occupation*				
Nurse/midwife/feldsher	3932 (71.7)	1555 (28.3)	1 (Reference)	1 (Reference)
Other medical	3091 (74.1)	1078 (25.9)	0.88 (0.80–0.97)**	0.91 (0.83–1.00)
Non‐medical/director/ manager	2821 (70.6)	1176 (29.4)	1.05 (0.96–1.15)	1.06 (0.97–1.17)
*Level of health organization*				
Primary	1413 (75.6)	457 (24.4)	1 (Reference)	1 (Reference)
Secondary	5214 (72.6)	1971 (27.4)	1.17 (1.04–1.31)**	1.15 (1.02–1.30)*
Tertiary	3013 (69.7)	1310 (30.3)	1.34 (1.19–1.52)***	1.31 (1.15–1.49)***
Health department	204 (74.2)	71 (25.8)	1.08 (0.80–1.44)	1.16 (0.86–1.56)
*Years in employment (years)*				
≤9	4383 (75.0)	1460 (25.0)	1 (Reference)	1 (Reference)
10–19	2373 (70.8)	978 (29.2)	1.24 (1.12–1.36)***	1.14 (1.02–1.27)*
20≤	3088 (69.3)	1371 (30.7)	1.33 (1.22–1.45)***	1.14 (0.99–1.30)
*Night shift*				
No	4788 (73.5)	1729 (26.5)	1 (Reference)	1 (Reference)
Yes	5056 (70.9)	2080 (29.1)	1.14 (1.06–1.23)**	1.13 (1.04–1.23)**

Abbreviations: AOR, adjusted odds ratio; CI, confidence interval; OR, odds ratio.

^a^ Adjusted for all variables listed in the table.

* *P* < 0.05

** *P* < 0.01‑

*** *P* < 0.001.

Costs of absenteeism among HCWs at all the 282 healthcare organizations from 2016 to 2018 were estimated and the average cost per absentee was calculated: the average lost salary was 34.7 USD, the average lost productivity was 129.8 USD, the average compensation was 130.9 USD, and the total average cost was 295.5 USD (Table 5). The average total cost in urban areas (312.9 USD) was higher than that in rural areas (271.6 USD), but the difference between the average cost between rural and urban areas was not significant in the primary (253.1 USD vs. 248.6 USD) and secondary (279.0 USD vs. 287.0 USD) levels. The average total cost was highest at healthcare organizations of the tertiary level (338.6 USD), which were all in urban areas, among all organization levels. The average compensation per absentee ranged from 102.1 USD at the primary level in urban areas to 149.4 USD at the tertiary level. The average compensation was 3.04–3.94 times higher than the average lost salary and the rate was lowest at the primary level in rural areas.

**TABLE 5:** Absenteeism costs of healthcare workers from 2016 to 2018 according to the levels of healthcare organizations and areas.

Level of organizations	Absentee (*n*)	Lost productivity (USD)		Lost salary (USD)		Compensation (USD)		Total cost (USD)	
		Total	Average^b^	Total	Average^b^	Total	Average^b^	Total	Average^b^
*Total*	13 653	1 772 805	129.8	473 951	34.7	1 787 470	130.9	4 034 226	295.5
Rural	5752	682 704	118.7	178 508	31.0	700 769	121.8	1 561 981	271.6
Urban	7901	1 090 101	138.0	295 443	37.4	1 086 701	137.5	2 472 245	312.9
*Primary level*									
Total	1870	204 020	109.1	57 096	30.5	210 064	112.3	471 180	252.0
Rural	1393	150 182	107.8	41 089	29.5	161 342	115.8	352 613	253.1
Urban	477	53 838	112.9	16 007	33.6	48 722	102.1	118 567	248.6
*Secondary level*									
Total	7185	893 613	124.4	234 431	32.6	900 720	125.4	2 028 764	282.4
Rural	4168	512 407	122.9	131 610	31.6	518 997	124.5	1 163 014	279.0
Urban	3017	381 206	126.4	102 821	34.1	381 723	126.5	865 750	287.0
*Tertiary level*									
Total (urban)^a^	4323	644 165	149.0	173 595	40.2	645 883	149.4	1 463 643	338.6
*Health department*									
Total	275	31 007	112.8	8829	32.1	30 802	112.0	70 638	256.9
Rural	191	20 114	105.3	5809	30.4	20 430	107.0	46 353	242.7
Urban	84	10 893	129.7	3020	36.0	10 372	123.5	24 285	289.1
*Estimation at all healthcare organizations in the public sector (per year)* ^c^									
Total	6078.2^d^	789 238	–	210 099	–	795 767	–	1 796 993	–

*Note*: 1USD = 2489 Mongolian Tugriks (MNT) in 2016, 2427 MNT in 2017, and 2644 MNT in 2018.

^a^ All healthcare organizations of the tertiary level located in Ulaanbaatar City.

^b^ Average per absentee.

^c^ The total number of healthcare organizations including family health centers was 654 in 2016, 652 in 2017, and 653 in 2018.

^d^ The estimated average number of absentees per year.

Each absenteeism cost at all 653 healthcare organizations in the public sector including 219 FHCs was estimated using the average cost. The total number of employees at all organizations was 28 976 in 2016, 29 572 in 2017, and 29 903 in 2018 and the average was 29 484. The average number of absentees was estimated to be 6078.2 (Table 5) based on the percentage of absentees to the total employees at 282 organizations (20.6%, Table 1). The estimated total cost at all organizations in the public sector per year was 1 796 993 USD including total lost productivity (789 238 USD), total lost salary (210 099 USD), and total compensation (795 767 USD) (Table 5).

## 4 DISCUSSION

In this study, the AR of HCWs in the public sector in Mongolia was 0.9%, which was lower compared with that in other countries. Previous studies demonstrated that the AR of HCWs was different depending on countries and kinds of professionals; 0.6%–1.1% at a teaching hospital in Iran^21^; 3.5% in the health sector of the UK, respectively^22^; and 2.0% at a teaching hospital in Brazil.^23^ According to the data of all HCWs in the UK, the AR was lowest among doctors (1.1%–1.3%) and the highest was among ambulance workers (5.5%) followed by nurses (4.5%).^22^ The reasons for the lower AR in this study may be due to the regulations on medically certified SA and low compensation. The maximum length of paid SA is 132 workdays for cancer and tuberculosis and 66 workdays for other diseases. The payment for paid sick leave is 50%–75% of the wage and the wage of HCWs is low, especially in the public sector of Mongolia. A shortage of nursing staff may also contribute to the low AR because nurses may feel pressure to work and therefore may not take SA. Previous studies reported that the number of sick days was lower and the attendance of sick workers was higher in countries where there was no or limited benefit of paid sick leave.^24^, ^25^

The top three causes of SA among HCWs were diseases of the genitourinary system, the digestive system, and the circulatory system, which were common diseases in the Mongolian population. The most common diseases in the population have been diseases of the respiratory system followed by diseases of the digestive system, diseases of the circulatory system, diseases of the genitourinary system, and injuries, poisoning, and certain other consequences of external causes since 2001.^10^, ^26^ The fifth common cause of SA among HCWs was diseases of the nervous system, such as dystonia, headache syndromes, and nerve root and plexus disorders. These may be caused stress by heavy workload or anxiety related to their work. Respiratory disease was the sixth common cause in this study, although the respiratory disease is the most common disease in the Mongolian population and the most common cause of SA among HCWs in other countries.^27^, ^28^, ^29^ Mongolian HCWs might have uncertified SA or work when they had minor respiratory diseases such as acute respiratory illness or influenza‐like illness. A previous study including employees who worked for the private sector in Ulaanbaatar reported that respiratory disease was the major cause of SA, especially during winter, but that most SA was uncertified.^9^

Factors associated with taking a long‐term absence among HCWs who had SA were the age group ≥40 years old, 10–19 years in employment compared with 9 years or less, working at the secondary and tertiary levels compared with the primary level, and having night shifts. Older age and longer experience were associated with having a long‐term SA. Previous studies reported that younger workers have more short‐term SA and more noncertified SA compared with older workers.^30^, ^31^, ^32^, ^33^, ^34^ Younger workers have fewer chronic diseases, can recover from illness and injury earlier, and are more likely to have absences due to minor health problems or motivational issues compared with older workers.^33^ In terms of the night shift, shift work negatively affects an employee's health and can increase the risk of cardiovascular diseases, gastrointestinal disorders, and cancer.^35^, ^36^, ^37^ It was suggested that the intensity or the number of consecutive night shifts was a risk factor for a long‐term SA,^38^, ^39^ although detailed schedules of shift work were not included in this study, such as rotation types, the number of consecutive night shifts or intensity, and years of shift work.

A review of studies on SA among HCWs reported that absenteeism and long‐term SA are more often at bigger and higher level organizations.^8^ HCWs of organizations in the secondary or tertiary level might be more exposed to physical and mental stress, because they work for more patients, perform complicated medical procedures, make more difficult decisions regarding patients' diseases, communicate with more other professionals, and have more night shifts compared with the primary health facilities. Another reason for more long‐term SA at the higher level may be because group cohesiveness is low and individual effort is unnoticed at large organizations.^8^ Long‐term SA is reported as a risk factor for permanent work disability.^40^ To reduce and manage long‐term SA, especially at large healthcare organizations in the public sector, increasing the motivation of HCWs to return to their work through early intervention by trained managers and robust implementation of SA policies are recommended.^41^, ^42^

The total cost due to SA in the public sector was estimated to be 1 796 993 USD per year, which accounted for 0.8% of the average annual expenditure of all healthcare organizations in the public sector (222 615 514 USD). However, this may be overestimated because the response rate at higher organizational levels was higher: 100.0% (16/16) at the tertiary level, 79.7% (47/59) at the secondary level, 37.2% (207/556) at the primary level, and 54.5% (12/22) at health departments. HCWs who worked for organizations at the higher level had more long‐term SA and their average absenteeism costs were higher than those who worked for the lower level. Therefore, the actual average absenteeism costs might be lower than the estimated costs in this study. However, the results of this study showed that the absenteeism cost at health organizations in the public sector is substantial, although the AR was low. According to the Labor Law, the working hours should be reduced when employees are diagnosed with occupational diseases or have an industrial accident but not general diseases based on the decision of the Occupational Health Center. The compensation and the maximum length of paid sick leave are regulated by the law, but the national guidelines for SA have not been developed yet. The low AR in this study may suggest high sickness presenteeism of HCWs, which would increase inefficient costs. Policymakers should evaluate the policy and regulations related to SA to make sure healthy life of HCWs in Mongolia.

There are some limitations to this study. First, this study included only medically certified SA but not noncertified SA or short‐term SA for 1–3 days. In Mongolia, employees of most public organizations are allowed to be absent from work for up to 3 days for any reason by their internal regulations. It is suggested that taking frequent short‐term SA is a risk factor for having a long‐term SA.^30^ Therefore, a further study on HCWs who have short‐term SA is needed because they should be targeted to reduce total SA days. Second, this study did not include all healthcare organizations in the public sector, because the response rate was 43.2%. However, 76.3% of HCWs in the public sector were included in this study. SA among HCWs in the private sector also needs to be studied, because 19.8–20.7% of all HCWs worked in the private sector in 2016–2018, although long‐term SA is reported to be more frequent in the public sector than in the private sector in some countries.^41^, ^43^, ^44^ Third, this study included only HCWs who had SA in 2016–2018 but did not include all HCWs. The routine health statistics includes only the number of employees according to sexes, age categories, and occupations. Therefore, in this study, the characteristics of HCWs cannot be compared between those who had SA and those who did not have SA.

## 5 CONCLUSIONS

To the best of our knowledge, this is the first study on SA among HCWs in Mongolia. The AR of HCWs in the public sector was 0.9% and the most common cause of certified SA was diseases of the genitourinary system. Factors associated with taking a long‐term SA among HCWs who had SA were 40 years old or older, 10–19 years in employment compared to 9 years or shorter, higher organizational level, and having night shifts. The average total cost per absentee was estimated to be 295.5 USD, and the total cost due to SA in the public sector was estimated to be 1 796 993 USD per year. To reduce costs due to SA and promote the well‐being and health of HCWs, policymakers should review the policy and regulations related to SA and develop national guidelines about SA for employers, healthcare managers, and absent HCWs in Mongolia.

## AUTHOR CONTRIBUTIONS

Erdenetuya Sukhee and Eiko Yamamoto conceived the ideas; Erdenetuya Sukhee and Ulzii‐Orshikh Khaltar collected the data; Erdenetuya Sukhee, Tugsdelger Sovd, Ulzii‐Orshikh Khaltar, and Eiko Yamamoto analyzed the data; Nobuyuki Hamajima, Enkhbold Sereenen, and Narantuya Davaakhuu provided technical support and interpretation of the data; and Erdenetuya Sukhee wrote the first draft of the manuscript. All authors revised the manuscript and approved the final version of the manuscript.

## CONFLICT OF INTEREST STATEMENT

The authors have no conflict of interest to disclose.

## Data Availability

The data that support the findings of this study are available from the Ministry of Health, Mongolia. Restrictions apply to the availability of these data, which were used under license for this study. Data are available from the authors with the permission of the Ministry of Health, Mongolia.

## FORMULAS FOR PARAMETERS OF SICKNESS ABSENCES

**Table:**

Parameter		Formula
(A)	Total number of employees	
(B)	Available workdays (days)	
(C)	Total available workdays of all employees (days)	(A) × (B)
(D)	Number of absentees	
(E)	Number of absences	
(F)	Total absence days	
(G)	Absence rate (%)	((F) × 5/7)/(C) × 100
(H)	Absence frequency rate (%)	(E)/(A) × 100
(I)	Average absence length (days)	(F)/(E)
(J)	Average absence length per absentee (days)	(F)/(D)
(K)	Average absence length per employee (days)	(F)/(A)
(L)	Average frequency of absences per absentee	(E)/(D)
